# Supplementary material for: Genome-wide association analyses identify two susceptibility loci for pachychoroid disease central serous chorioretinopathy
Source: Commun Biol. 2019 Dec 12;2:468. doi: 10.1038/s42003-019-0712-z (PMC6908630; doi:10.1038/s42003-019-0712-z)
Supplement: Supplementary file 1 — Supplementary Information [file 42003_2019_712_MOESM1_ESM.pdf]

Supplementary Figures

Supplementary Figure 1: Quantile-quantile (QQ) plots from the discovery stage. QQ plots for the association between all analyzed single-nucleotide polymorphisms and CSC in the discovery stage. Each blue dot represents an observed statistic (defined as the -log10(P-value)) versus the corresponding expected statistic before genomic control, whereas each red dot represents the observed statistic versus the corresponding expected statistic after genomic control. The black line corresponds to the null distribution. The genomic inflation factor lambda (λ_{GC}) was 1.157.

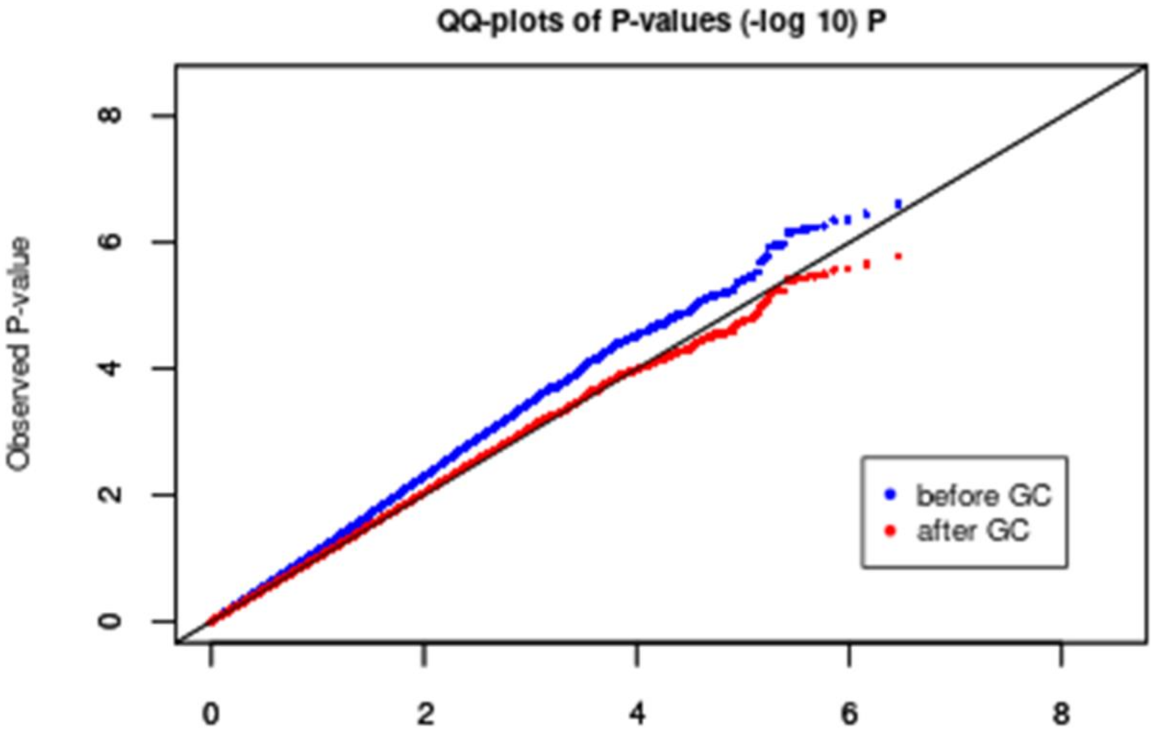

Supplementary Figure 2. Principal component analysis using five populations (AFR, SAS, EUR, EAS, JPT) from 1000 Genomes project and samples in the discovery GWAS (current study). The scatter plots for the first and second principal components.

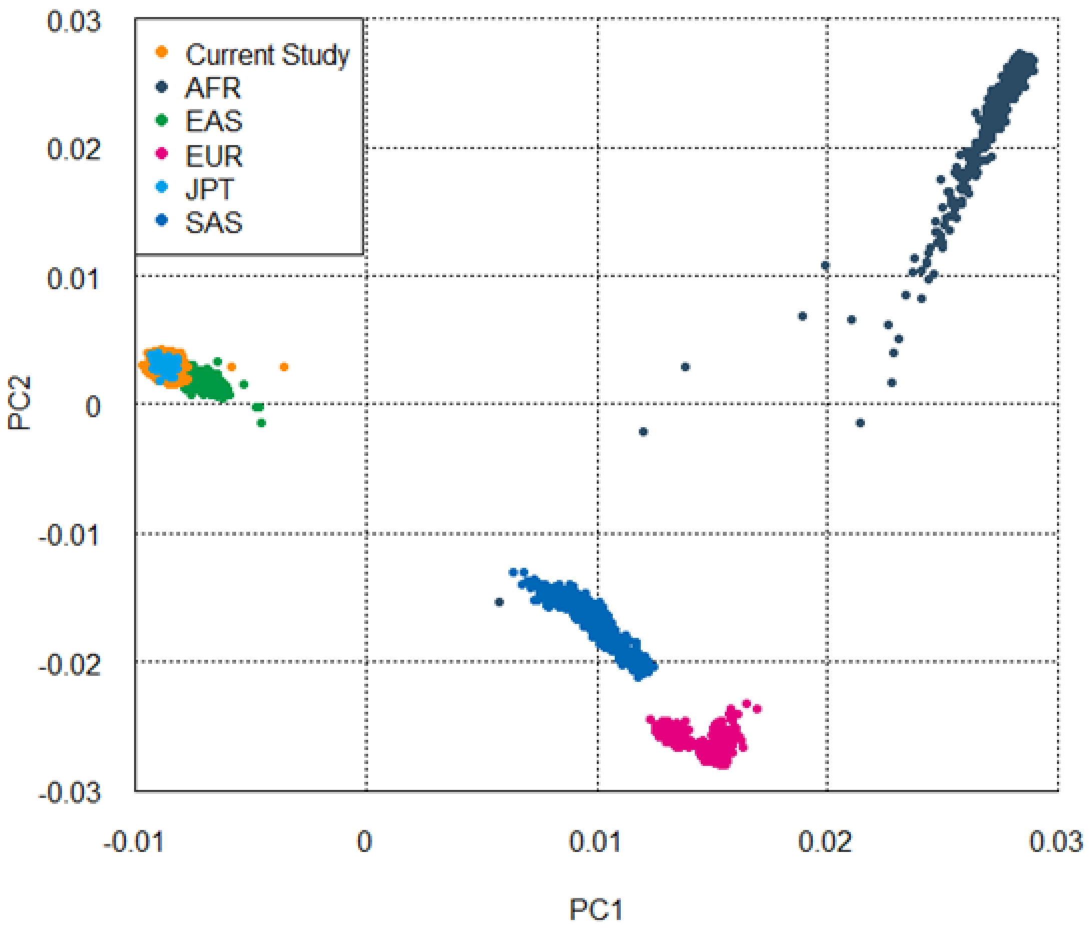

Supplementary Figure 3. Results of the principal component analysis using Japanese samples in the discovery GWAS. The scatter plots for the (A) first and second principal components, and (B) the second and third components.

Black and orange dots correspond to CSC and control samples, respectively.

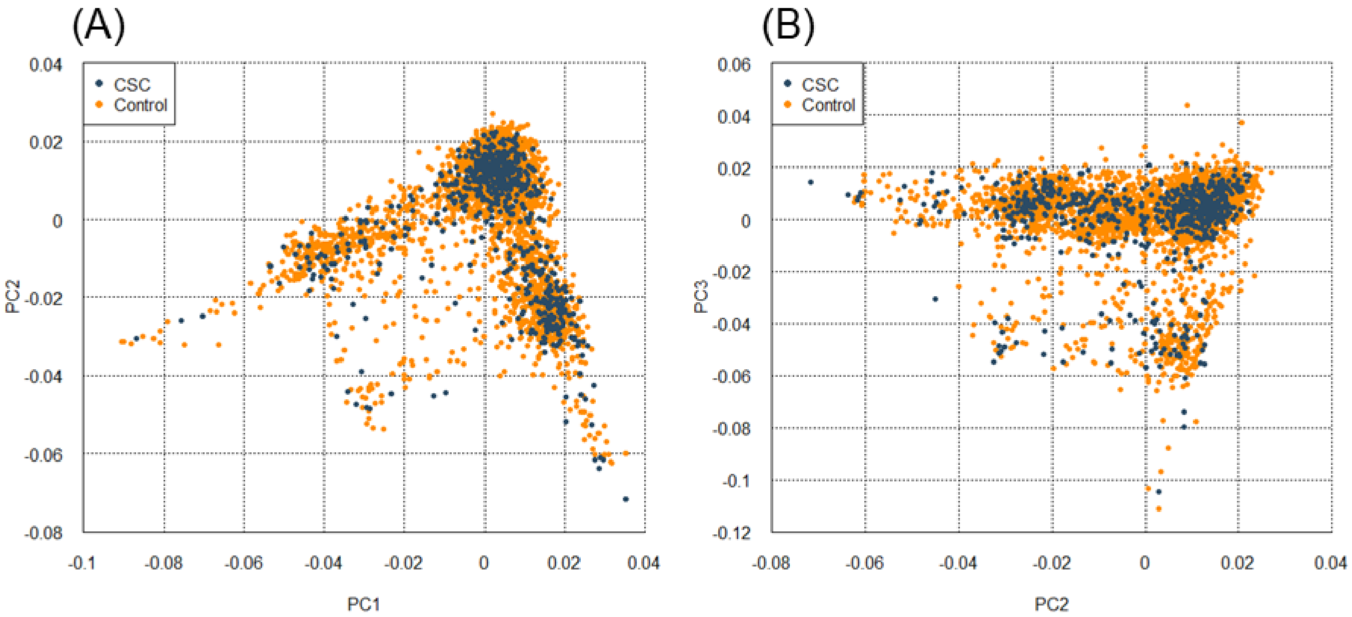

Supplementary Figure 4. Case-control association result and linkage disequilibrium (LD) map of the *TNFRSF10A-LOC389641* region.

LD block was defined based on the confidence interval rule.

Plots represent the −log10(P-values) obtained from the first-stage GWAS. The LD map based on R^2 values was drawn using genotype data of the cases and controls in GWAS samples.

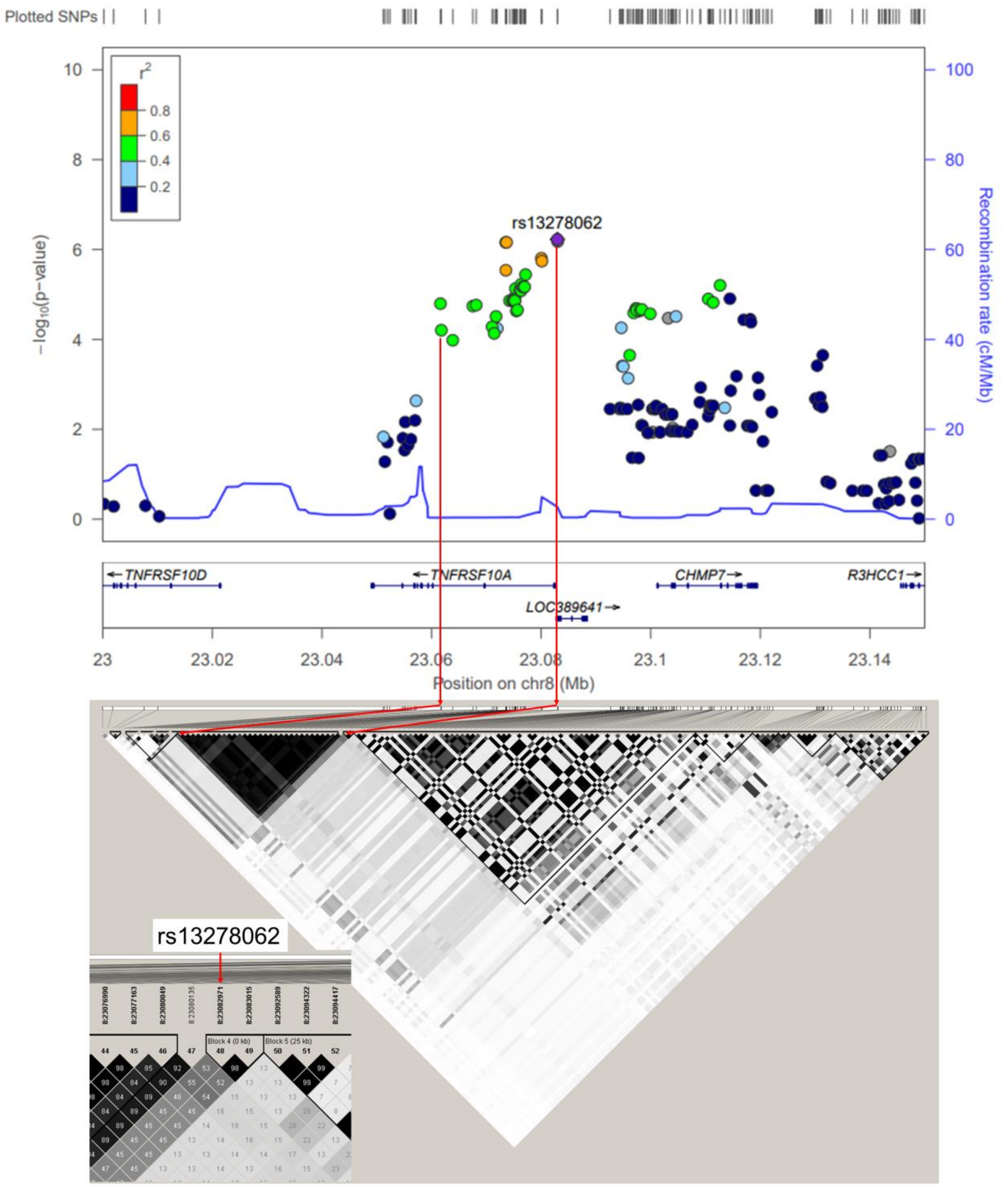

Supplementary Figure 5: Regional association plots around *GATA5* regions using GWAS data without quality control.

A strong signal is observed within the *GATA5* region; rs13044490 exceeded the genome-wide significance threshold ($P = 2.94 \times 10^{-10}$). The odds ratio for the occurrence of CSC was 1.67 (95% confidence interval, 1.42–1.95).

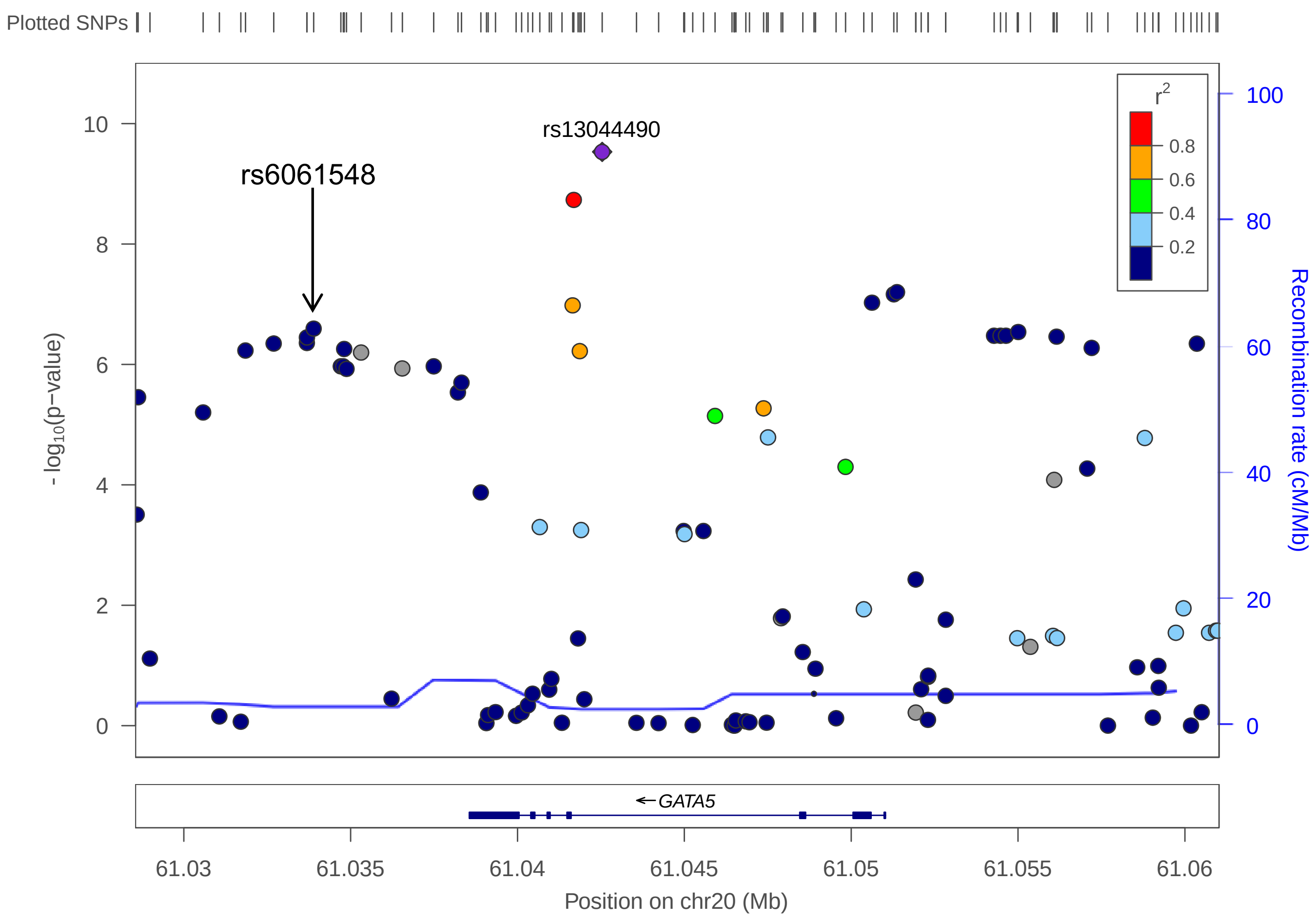

Supplementary Figure 6. Multi-tissue eQTL comparison of association between rs13278062 and *TNFRSF10A* expression.

Multi-tissue eQTL plot from publicly available quantitative trait locus analysis (eQTL) database search (GTEx Portal. https://gtexportal.org/home/) is shown. The effect size of rs13278062 on *TNFRSF10A* expression was strongest in the adrenal gland (normalized effect size = -0.973, $P = 4.5 \times 10^{-39}$), followed by the aorta (normalized effect size = -0.827, $P = 1.6 \times 10^{-44}$).

Tissue	Samples	NES	p-value	m-value
Pancreas	220	0.0873	0.2	0.00
Esophagus - Mucosa	358	-0.0400	0.1	0.00
Brain - Cerebellum	154	-0.0623	0.4	0.00
Liver	153	-0.0698	0.3	0.00
Brain - Cerebellar Hemisphere	125	-0.0707	0.4	0.0210
Brain - Frontal Cortex (BA9)	118	-0.0714	0.4	0.0160
Brain - Putamen (basal ganglia)	111	-0.0895	0.3	0.0430
Vagina	106	-0.0910	0.3	0.104
Uterus	101	-0.0912	0.3	0.112
Skin - Sun Exposed (Lower leg)	414	-0.0975	6.0e-4	0.00
Brain - Hypothalamus	108	-0.107	0.2	0.0440
Brain - Anterior cingulate cortex (BA24)	109	-0.113	0.2	0.207
Skin - Not Sun Exposed (Suprapubic)	335	-0.120	9.4e-4	0.00
Brain - Cortex	136	-0.127	0.07	0.153
Brain - Substantia nigra	80	-0.129	0.2	0.544
Pituitary	157	-0.152	0.01	0.482
Minor Salivary Gland	85	-0.153	0.05	0.560
Brain - Nucleus accumbens (basal ganglia)	130	-0.158	0.06	0.569
Brain - Spinal cord (cervical c-1)	83	-0.164	0.1	0.693
Colon - Transverse	246	-0.172	3.9e-8	0.887
Breast - Mammary Tissue	251	-0.184	1.1e-5	1.00
Brain - Hippocampus	111	-0.189	0.01	0.966
Small Intestine - Terminal Ileum	122	-0.193	2.1e-4	1.00
Stomach	237	-0.194	9.8e-6	1.00
Muscle - Skeletal	491	-0.200	6.9e-8	1.00
Brain - Caudate (basal ganglia)	144	-0.206	4.3e-3	0.968
Prostate	132	-0.206	1.4e-3	1.00
Brain - Amygdala	88	-0.218	2.0e-3	1.00
Thyroid	399	-0.228	2.3e-9	1.00
Testis	225	-0.235	2.3e-4	1.00
Artery - Coronary	152	-0.244	6.6e-6	1.00
Nerve - Tibial	361	-0.247	1.2e-8	1.00
Ovary	122	-0.251	7.0e-5	1.00
Heart - Left Ventricle	272	-0.257	1.5e-8	1.00
Esophagus - Gastroesophageal Junction	213	-0.259	2.4e-5	1.00
Cells - EBV-transformed lymphocytes	117	-0.264	0.02	0.996
Whole Blood	369	-0.279	2.7e-13	1.00
Artery - Tibial	388	-0.304	2.2e-23	1.00
Lung	383	-0.308	1.0e-18	1.00
Adipose - Subcutaneous	385	-0.331	1.5e-23	1.00
Colon - Sigmoid	203	-0.378	3.5e-11	1.00
Adipose - Visceral (Omentum)	313	-0.391	2.4e-21	1.00
Heart - Atrial Appendage	264	-0.396	7.5e-14	1.00
Esophagus - Muscularis	335	-0.416	5.5e-22	1.00
Spleen	146	-0.491	2.2e-11	1.00
Cells - Transformed fibroblasts	300	-0.666	4.3e-58	1.00
Artery - Aorta	267	-0.827	1.6e-44	1.00
Adrenal Gland	175	-0.973	4.5e-39	1.00

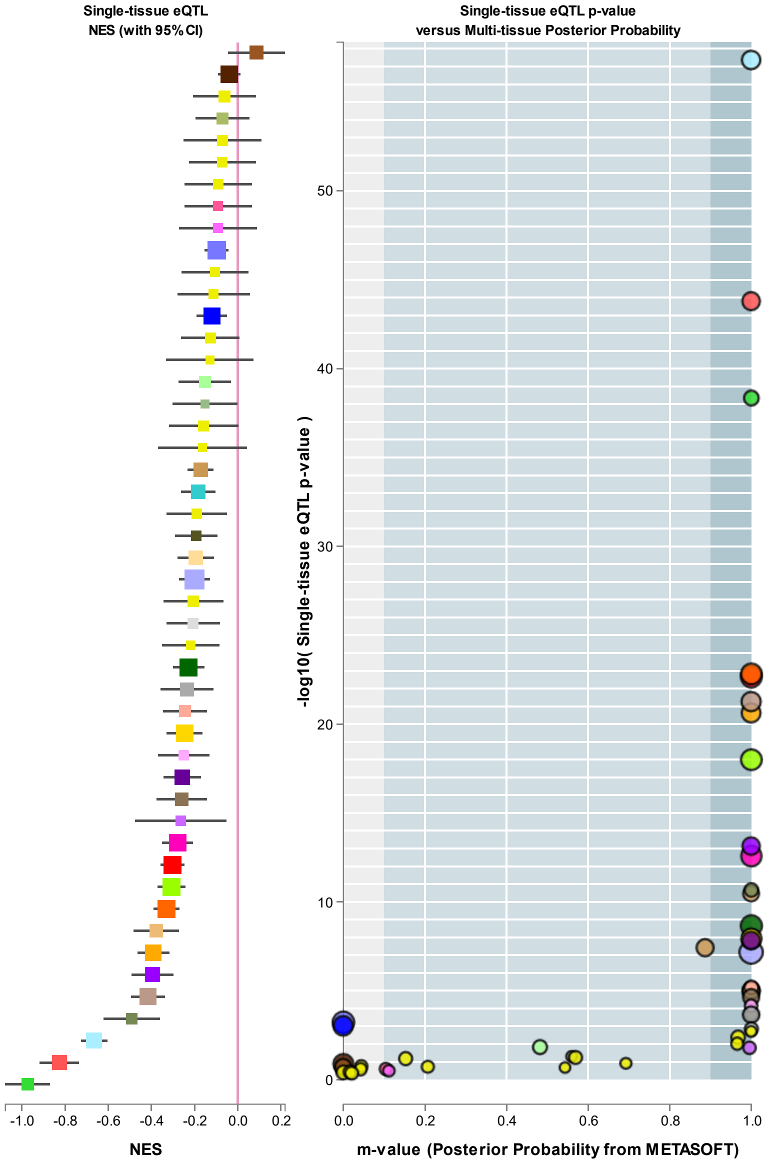

Supplementary Figure 7. Multi-tissue eQTL comparison of association between rs13044490 and *GATA5* expression.

Multi-tissue eQTL plot from publicly available quantitative trait locus analysis (eQTL) database search (GTEx Portal. https://gtexportal.org/home/) is shown. The effect size of rs13044490 on *GATA5* expression was the strongest in sun-exposed skin (normalized effect size = 0.353, $P = 3.8 \times 10^{-7}$) and esophageal muscularis (normalized effect size = 0.347, $P = 3.9 \times 10^{-8}$).

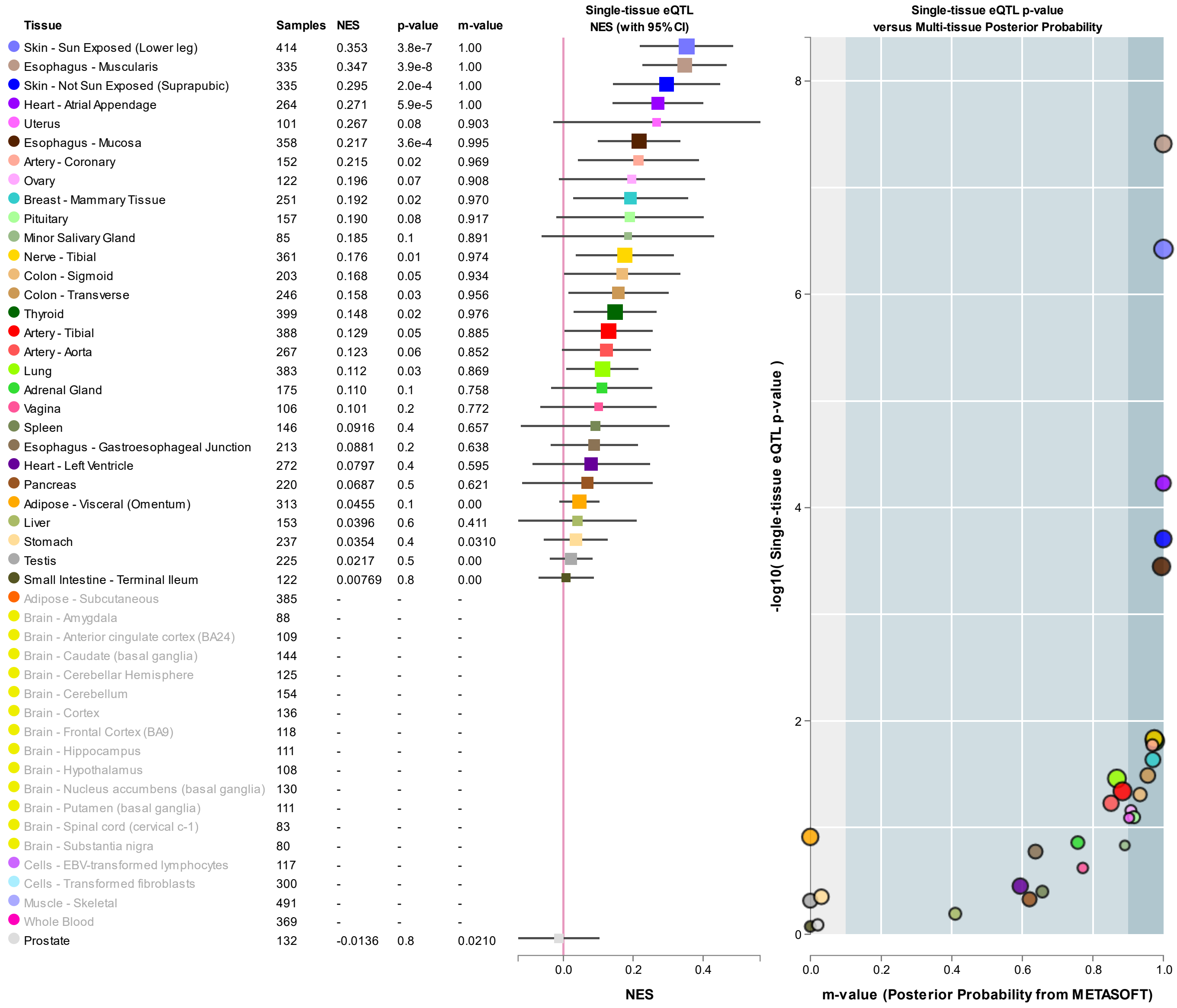

Supplementary Table 1. Description and test statistics of top 10 pathways from pathway analysis using VEGAS2

Pathway ID	Pathway length	Nominal *P* value	Empirical *P* value	Genes
M00412_ESCRT-III_complex	10	1.57×10^{-7}	2.60×10^{-5}	RNF103-CHMP3_CHMP2B_CHMP7_CHMP4C_CHMP5_CHMP4A_CHMP6_CHMP2A_CHMP4B
GO:0000920_cell_separation_after_cytokinesis	16	2.19×10^{-7}	0.00012	RNF103-CHMP3_MITD1_PDCD6IP_CHMP2B_CHMP7_CHMP4C_CHMP5_CEP55_CHMP4A_VPS4A_CHMP1A_CHMP6_VPS4B_CHMP2A_CHMP4B
GO:0046755_viral_budding	23	7.90×10^{-7}	0.00031	RNF103-CHMP3_MITD1_PDCD6IP_CHMP2B_VTA1_VPS37D_VPS37A_CHMP7_CHMP4C_VPS28_CHMP5_LRSAM1_TSG101_VPS37B_CHMP4A_VPS4A_CHMP1A_CHMP6_VPS4B_MVB12A_CHMP2A_CHMP4B
GO:1902592_multi-organism_membrane_budding	23	7.90×10^{-7}	0.00033	RNF103-CHMP3_MITD1_PDCD6IP_CHMP2B_VTA1_VPS37D_VPS37A_CHMP7_CHMP4C_VPS28_CHMP5_LRSAM1_TSG101_VPS37B_CHMP4A_VPS4A_CHMP1A_CHMP6_VPS4B_MVB12A_CHMP2A_CHMP4B
GO:0036258_multivesicular_body_assembly	28	7.65×10^{-7}	0.0005	RNF103-CHMP3_STAM2_PDCD6IP_CHMP2B_VTA1_VPS37D_VPS37A_CHMP7_CHMP4C_VPS28_CHMP5_STAM_TSG101_VPS37B_VPS36_CHMP4A_RAB11A_VPS4A_IST1_CHMP1A_SNF8_CHMP6_HGS_VPS4B_MVB12A_CHMP2A_CHMP4B
GO:1902590_multi-organism_organelle_organization	23	7.90×10^{-7}	0.0005	RNF103-CHMP3_MITD1_PDCD6IP_CHMP2B_VTA1_VPS37D_VPS37A_CHMP7_CHMP4C_VPS28_CHMP5_LRSAM1_TSG101_VPS37B_CHMP4A_VPS4A_CHMP1A_CHMP6_VPS4B_MVB12A_CHMP2A_CHMP4B
GO:0045947_negative_regulation_of_translational_i	18	2.47×10^{-6}	0.00052	EIF2B3_TPR_EIF2B4_PAIP2B_EIF2AK3_EIF2B5_BANK1_PAIP2_EIF4EBP3_EIF2AK1_EIF3E_AGO2_EIF4EBP2_RBM4_EIF2S1_EIF2A

nitiation				K4_RARA_RPL13A
GO:0044803_multi-organism_membrane_organization	26	1.68×10^{-6}	0.00054	RNF103-CHMP3_MITD1_PDCD6IP_HYAL2_CHMP2B_VTA1_VPS37D_VPS37A_CHMP7_CHMP4C_VPS28_CHMP5_LRSAM1_TSG101_VPS37B_GAS6_CHMP4A_VPS4A_CHMP1A_CHMP6_VPS4B_MVB12A_PVRL2_CHMP2A_CHMP4B
R-HSA-162588_Budding_and_maturation_of_HIV_virion	22	2.30×10^{-6}	0.00058	RPS27A_CHMP3_PDCD6IP_CHMP2B_VTA1_VPS37D_VPS37A_CHMP7_CHMP4C_VPS28_CHMP5_TSG101_VPS37B_UBC_CHMP4A_VPS4A_CHMP6_NEDD4L_VPS4B_UBA52_CHMP2A_CHMP4B
GO:0036257_multivesicular_body_organization	29	1.18×10^{-6}	0.0006	RNF103-CHMP3_STAM2_PDCD6IP_CHMP2B_VTA1_VPS37D_VPS37A_CHMP7_CHMP4C_VPS28_CHMP5_STAM_TSG101_VPS37B_VPS36_CHMP4A_RAB27A_RAB11A_VPS4A_IST1_CHMP1A_SNF8_CHMP6_HGS_VPS4B_MVB12A_CHMP2A_CHMP4B

Supplementary Table 2. Discovery and meta-analyses of rs13278062 and rs6061548 with CSC after genomic control correction.

SNP	rs13278062		rs6061548	
	Sample size	P value	Sample size	P value
Discovery GWAS (before GC correction)	3,460	5.94×10^{-7}	3,460	2.52×10^{-7}
Discovery GWAS (after GC corrected)	3,460	1.59×10^{-5}	3,460	8.34×10^{-6}
Meta-analysis (after GC correction)	14,574	6.29×10^{-12}	13,672	2.57×10^{-12}

Supplementary Table 3. Summary of the studied cohorts.

		CSC			Controls		
Stage	Ethnicities	Institutes	N	Genotyping platform	Institutes	N	Genotyping platform
Discovery GWAS	Japanese	Kyoto	610	Omni Express, Asian Screening Array	Aichi Cancer Center Research Institute, Hayashi Eye Hospital, Mizoguchi Eye Hospital, Oita, Ideta Eye Hospital, Shinjo Eye Clinic, Miyata Eye Hospital, Ozaki Eye Hospital, Kyoto, Nagahama City Hospital[1]	2,850	Human610-Quad BeatChip, Omni Express
Replication stage 1	Japanese	Kyoto, Kagawa, Yamanashi, Fukushima	288	TaqMan genotyping assay	Kyoto University,[5,6] Tohoku dataset,[2–4] Yokohama City University dataset	5,449	Omni Express
Replication stage 2	Japanese	Kobe datasets[7]	137	Omni Express	Kobe datasets[7]	1,153	Omni Express
Replication stage 3	Caucasian	Caucasian dataset (Nijmegen, Cologne, Leiden)[8]	521	Omni Express	Caucasian dataset (NBS)[8]	3,577	Omni Express

References

1. Aung, T. *et al.* A common variant mapping to CACNA1A is associated with susceptibility to exfoliation syndrome. *Nat. Genet.* **47,** 387-392 (2015).
2. Kuriyama, S. *et al.* The Tohoku Medical Megabank Project: design and mission. *J. Epidemiol.* **26,** 493-511 (2016).
3. Yamaguchi-Kabata, Y. *et al.* iJGVD: an integrative Japanese genome variation database based on whole-genome sequencing. *Hum. Genome Var.* **2,** 15050 (2015).
4. Nagasaki, M. *et al.* Rare variant discovery by deep whole-genome sequencing of 1,070 Japanese individuals. *Nat. Commun.* **6,** 8018 (2015).
5. Higasa, K. *et al.* Human genetic variation database, a reference database of genetic variations in the Japanese population. *J. Hum. Genet.* **61,** 547-553 (2016).
6. Narahara, M. *et al.* Large-scale East-Asian eQTL mapping reveals novel candidate genes for LD mapping and the genomic landscape of transcriptional effects of sequence variants. *PLoS One* **9,** e100924 (2014).
7. Miki, A. *et al.* Genome-wide association study to identify a new susceptibility locus for central serous chorioretinopathy in the Japanese population. *Investig. Ophthalmology Vis. Sci.* **59,** 5542 (2018).
8. Schellevis, R. L. *et al.* Role of the complement system in chronic central serous chorioretinopathy: a genome-wide association study. *JAMA Ophthalmol.* (2018). doi:10.1001/jamaophthalmol.2018.3190

Supplementary Note

Description of the cohorts and genotyping

Discovery GWAS

CSC patients (n = 610) were recruited from Kyoto University Hospital. The diagnosis was made as described in the Methods section. For controls, existing genome-wide datasets were utilized. The data for the healthy Japanese cohort (n = 2,850) were drawn from eight institutions across Japan (Aichi Cancer Center Research Institute, Hayashi Eye Hospital in Fukuoka, Mizoguchi Eye Hospital in Nagasaki, Department of Ophthalmology, Oita University Faculty of Medicine, and four sites in Miyazaki: Ideta Eye Hospital, Shinjo Eye Clinic, Miyata Eye Hospital, and Ozaki Eye Hospital, Kyoto University Hospital, and Nagahama City Hospital). Although detailed ophthalmic examinations were not performed for healthy Japanese subjects from the Aichi Cancer Center Research Institute (n = 1,194), the other 1,656 samples were confirmed to not have exfoliation syndrome, macular degeneration, or glaucoma, as described previously.[1] A series of BeadChip DNA arrays (Illumina, San Diego, CA, USA), namely Omni Express (n = 250) and Asian Screening Array (n = 360) were used for genotyping the CSC patients, and Human610-Quad BeatChip (n = 1,194) and Omni Express (n = 1,656) were used for genotyping of the control samples. SNPs with a call

rate <90% or minor allele frequency (MAF) <1% were excluded, and genotype imputation was performed using the Michigan imputation server (https://imputationserver.sph.umich.edu/index.html#!pages/home) with the 1000 Genomes dataset (phase3 v5 release) of East Asian subjects as a reference panel, and Eagle v2.3 was used as phasing software. Quality control was again performed for each platform after imputation; SNPs with a call rate <90%, MAF <1%, significant deviation ($P < 1.0 \times 10^{-5}$) from Hardy–Weinberg equilibrium, or call rate <90% were excluded from further analyses. We evaluated the allelic discrimination of SNPs showing a suggestive association with CSC in the discovery GWAS ($P < 1.0 \times 10^{-5}$) for each platform. We excluded SNPs with an insufficient quality of allelic discrimination and their proxy SNPs ($R^2 > 0.8$). Finally, 2,893,743 SNPs from 610 CSC samples and 2,850 control samples were used for discovery stage analysis.

Replication Stage 1

CSC cohort

For the replication stage, additional patients with CSC (n = 278) were recruited from across Japan (Kyoto University Hospital, Kagawa University Hospital, Yamanashi University Hospital, and the Fukushima Medical University Hospital). The diagnosis was made by ophthalmologists at each institute based on dilated fundus examination, optical coherence

tomography, and/or fluorescein and indocyanine green angiography. Genotypes were determined using a commercially available TaqMan SNP assay (Applied Biosystems, Foster City, CA, USA). Deviation from Hardy-Weinberg Equilibrium was assessed using R software.

Integrative Japanese Variation Database (Tohoku Dataset)

The Integrative Japanese Genome Variation Database (ver 3.5K JPN, https://ijgvd.megabank.tohoku.ac.jp/) provides genomic reference panels obtained from 3,554 normal Japanese subjects. Details of this cohort are described elsewhere.[2–4] Briefly, samples were recruited from the Tohoku Medical Megabank Organization, Iwate Medical Megabank Organization, Nagahama Prospective Cohort for Comprehensive Human Bioscience, and National Hospital Organization Nagasaki Medical Center. All DNA samples were whole genome-sequenced using the Illumina HiSeq 2500. This dataset contains the allele frequency data for 37,067,715 reliable autosomal single-nucleotide variations (SNVs) detected by whole-genome sequencing of 3,552 Japanese individuals (3.5KJPN release September 28, 2017). We used the dataset of 7,931,579 SNVs with more than or equal to 1% of the Japanese population allele frequency. Genotypes of rs13278062 and rs6061548 were available.

Human Genetic Variation Database (Kyoto University)

The Human Genetic Variation Database is a database of genomic reference panels released from Kyoto University (http://www.hgvd.genome.med.kyoto-u.ac.jp/index.html). The details of this database are described elsewhere.[5,6] Briefly, this database is a web-accessible resource of genetic variations in the Japanese population and contains 1,794,196 variants of 3,248 healthy individuals and 287,588 SNVs additionally identified by whole-exome sequencing of 1,208 healthy individuals. Whole-genome SNV genotyping was performed for 3,712 individuals, who formed a subset of participants of The Nagahama Prospective Genome Cohort for the Comprehensive Human Bioscience (the Nagahama Study), with the Illumina HumanHap610 quad, Omni 2.5M and Human exome Beadarrays (Illumina). After excluding samples for which the genotyping call rates were lower than 95%, kinship analysis and principal component analysis were applied. A total of 302 related individuals were excluded from further analysis, resulting in a dataset of 3,248 East Asian individuals. SNPs with <99% genotyping success rates, with minor allele frequencies lower than 0.01, or with Hardy Weinberg equilibrium *P*-values lower than 1×10^{-7} were excluded. Additionally, exomic sequencing data of 1,208 Japanese individuals from five institutes, including Kyoto University, National Research Institute for Child Health and Development, Tohoku University, The University of Tokyo, and Yokohama City University, were available in this database. Exomic sequencing data were obtained using commercially available oligonucleotide libraries followed by applications to next-generation sequencers HiSeq1000 (Illumina),

HiSeq2000 (Illumina), and SOLiD 5500XL (Thermo Fisher Scientific, Waltham, MA, USA)). The genotypes of rs13278062 were obtained on the basis of the whole-genome SNP genotyping results, whereas the genotypes of rs6061548 were not available in this database.

Yokohama City University dataset

The Yokohama City University dataset includes 1,048 Japanese healthy controls recruited from the Yokohama City University, Okada Eye Clinic, and Aoto Eye Clinic in Yokohama, Kanagawa Prefecture, Japan. Genotypes of samples from Yokohama City University were determined using BeadChip DNA arrays, namely Human OmniExpress chip (Illumina), with the standard protocol recommended by each manufacturer. Samples with a call rate less than 97% were excluded. SNPs were excluded based on the following quality control criteria: call rate <98%; the rates of missing data were significantly different between cases and controls ($P < 1.0 \times 10^{-6}$); overall minor allele frequency <1%; and significant deviation from Hardy-Weinberg equilibrium in controls ($P < 1.0 \times 10^{-5}$). Additionally, cryptic relatedness between samples was estimated based on identity by descent; closely related samples with a pi-hat >0.1875 were eliminated. Finally, 556,905 autosomal SNPs (1,048 controls) on the Illumina Human OmniExpress chip that passed the filters were used for subsequent imputation analyses. The Michigan imputation server (https://imputationserver.sph.umich.edu/index.html#!pages/home) with the 1000 Genomes

dataset (phase3 v5 release) was used as a reference panel. All imputed SNPs were filtered with the quality control parameters (minor allele frequency >0.01 and squared correlation between imputed and true genotypes [r^2] > 0.7).

Replication stage 2 (Kobe CSC case-control dataset)

The details of this dataset are described elsewhere.[7] Briefly, individuals with idiopathic CSC recruited at Kobe University Hospital and population-based volunteers recruited by Kyushu University were used. Patients with idiopathic CSC, which represents central serous retinal detachment without subretinal hemorrhage or suspected choroidal neovascularization in ICGA or OCT, were included. Patients administered corticosteroid therapy, whose central choroidal thickness was less than 250 µm, who were aged over 80 years, and those with past histories of retinal vessel occlusion or uveitis were excluded. No ophthalmic evaluations were performed in control samples. Genotypes of samples were determined using BeadChip DNA arrays, namely Human Omni Express BeadChips (Illumina). Strand check and flipping to forward strand were performed using conform-gt (https://faculty.washington.edu/browning/conform-gt.html), the utility program for BEAGLE 4.1. Genotype data of 1000 Genomes CHB and JPT (The 1000 Genomes Project Consortium 2015a and 2015b) were used as references for the strand check procedure. Imputation was performed using BEAGLE 4.1 with genotype data of 1000 Genomes phase 3

(http://bochet.gcc.biostat.washington.edu/beagle/1000_Genomes_phase3_v5a/) as a reference panel. SNPs with an allelic R^2 lower than 0.8, call rate <95%, minor allele frequency <1% or significant deviation ($P < 1.0 \times 10^{-5}$) from Hardy–Weinberg equilibrium were excluded. Samples with a call rate <90% or pi-hat value > 0.25 were excluded from further analyses. Finally, 6,598,085 SNPs from 137 CSC samples and 1,153 controls were included in the dataset.

Replication stage 3 (Caucasian CSC case-control dataset)

The details of this dataset are described elsewhere.[8] Briefly, European patients with chronic CSC recruited from outpatient clinics at the Radboud University Medical Centre (N = 307), University Hospital of Cologne (N = 71), and Leiden University Medical Center (N = 143) were included. Patients included in this study showed the presence of serous fluid on optical coherence tomography in either eye, RPE irregularities with 1 or more hot spots of leakage on fluorescein angiography in either eye, and corresponding hyper fluorescence on indocyanine green angiography. Patients in whom evidence of another explanatory diagnosis or complication was present were excluded from this study. Controls were obtained from the Nijmegen Biomedical Study (NBS), a population-based survey conducted by the Department for Health Evidence and the Department of Laboratory Medicine of the Radboudumc. In the NBS, 21,756 randomly selected age- and gender-stratified inhabitants of the municipality of

Nijmegen were invited to complete a postal questionnaire on, e.g., lifestyle and medical history, and to donate an 8.5-mL blood sample in a serum separator tube and a 10-mL EDTA blood sample. In this population-based study, no ophthalmologic grading was performed.

Genotypes of 521 CSC patients were obtained using OmniExpress-12 or OmniExpress-24chip, and 3,577 controls for which genotyping was available on the Omni The express platform was included in the analysis. Quality control steps were applied to the separate batches using PLINK software. Samples with a call rate of <97% were removed. In each batch, SNPs with genotype call rates <98% or showing deviations from Hardy-Weinberg equilibrium ($P < 10^{-6}$) were excluded and only variants with a MAF >1% were retained. Only variants with a call rate >98% in the full dataset were preserved, leaving 589,487 autosomal and 13,282 X-chromosomal variants that could be used for downstream analysis. To assess population stratification, the dataset was merged with the Hapmap3 data on individuals of known genetic ancestry. Data were pruned with a window size of 50 kb, step size of 5, and variance inflation factor of 2; principal component analysis was performed with PLINK. Only individuals of European ancestry were retained for further analysis. Cryptic relatedness within the dataset was analyzed with KING (v2.0). A kinship coefficient threshold of <0.0884 was used to remove duplicates and individuals with a first or second-degree relationship from the dataset. After quality control, a total of 589,487 autosomal and 13,282 X-chromosomal

variants were used to impute the dataset. Autosomal genotype data were phased using Eagle (v2.3), while the X chromosome was phased with ShapeIT (v2. r790). After phasing, the data were imputed with the Haplotype Reference Consortium release 1.1.2016 using the Michigan Imputation server (https://imputationserver.sph.umich.edu). SNPs were filtered on an imputation quality score of $R^2 > 0.3$ for common variants (MAF >5 %) and a $R^2 > 0.8$ for low frequency variants (MAF < 5%).

Expression of genes in human tissue

The Eyeintegration database (https://eyeintegration.nei.nih.gov/, v1.01) revealed that *TNFRSF10A* and *GATA5* are expressed in other human tissues (http://eyeIntegration.nei.nih.gov/?Dataset=Gene_2019&ID=TNFRSF10A,GATA5&Tissue=_Adipose_-_Subcutaneous_,_Adipose_-_Visceral_(Omentum)_,_Adrenal_Gland_,_Artery_-_Aorta_,_Artery_-_Coronary_,_Artery_-_Tibial_,_Bladder_,_Brain_-_Amygdala_,_Brain_-_Anterior_cingulate_cortex_(BA24)_,_Brain_-_Caudate_(basal_ganglia)_,_Brain_-_Cerebellar_Hemisphere_,_Brain_-_Cerebellum_,_Brain_-_Cortex_,_Brain_-_Frontal_Cortex_(BA9)_,_Brain_-_Hippocampus_,_Brain_-_Hypothalamus_,_Brain_-_Nucleus_accumbens_(basal_ganglia)_,_Brain_-_Putamen_(basal_ganglia)_,_Brain_-_Spinal_cord_(cervical_c-1)_,_Brain_-_Substantia_nigra_,_Breast_-_Mammary_Tissue_,_Cells_-_EBV-transformed_lymphocytes_,_Cells_-_Leukemia_cell_line_(CML)_,_Cervix_-_Ectocervix_,_Cells_-_Transformed_fibro

blasts_,_Cervix_-_Endocervix_,_Colon_-_Sigmoid_,_Colon_-_Transverse_,_Esophagus_-_Gastroesophageal_Junction_,_Esophagus_-_Mucosa_,_Esophagus_-_Muscularis_,_Fallopian_Tube_,_Heart_-_Atrial_Appendage_,_Heart_-_Left_Ventricle_,_Kidney_-_Cortex_,_Liver_,_Lung_,_Minor_Salivary_Gland_,_Muscle_-_Skeletal_,_Nerve_-_Tibial_,_Ovary_,_Pancreas_,_Pituitary_,_Prostate_,_Skin_-_Not_Sun_Exposed_(Suprapubic)_,_Skin_-_Sun_Exposed_(Lower_leg)_,_Small_Intestine_-_Terminal_Ileum_,_Spleen_,_Stomach_,_Testis_,_Thyroid_,_Uterus_,_Vagina_,_Whole_Blood_,Choroid_Plexus_-_Adult_Tissue,Cornea_-_Adult_Tissue,Cornea_-_Cell_Line_Endothelium,Cornea_-_Endothelium,Cornea_-_Fetal_Endothelium,Cornea_-_Limbus,Cornea_-_Stem_Cell_Endothelium,Cornea_-_Stroma,ESC_-_Stem_Cell_Line,EyeLid_-_Adult_Tissue,Lens_-_Stem_Cell_Line,Retina_-_3D_Organoid_Stem_Cell,Retina_-_Adult_Tissue,Retina_-_Adult_Tissue_AMD_MGS_2,Retina_-_Adult_Tissue_AMD_MGS_3,Retina_-_Adult_Tissue_AMD_MGS_4,Retina_-_Adult_Tissue_MGS_1,Retina_-_Fetal_Eye,Retina_-_Fetal_Tissue,Retina_-_RGC_Stem_Cell,Retina_Fetal_Tissue,Retinal_Endothelium_-_Adult_Tissue,RPE_-_Adult_Tissue,RPE_-_Cell_Line,RPE_-_Fetal_Tissue,RPE_-_Stem_Cell_Line&num=2, accessed 7 October 2019).

References

1. Aung, T. *et al.* A common variant mapping to CACNA1A is associated with susceptibility to exfoliation syndrome. *Nat. Genet.* **47,** 387-392 (2015).

2. Kuriyama, S. *et al.* The Tohoku medical megabank project: Design and mission. *J. Epidemiol.* **26,** 493-511 (2016).

3. Yamaguchi-Kabata, Y. *et al.* iJGVD: an integrative Japanese genome variation database based on whole-genome sequencing. *Hum. Genome Var.* **2,** 15050 (2015).

4. Nagasaki, M. *et al.* Rare variant discovery by deep whole-genome sequencing of 1,070 Japanese individuals. *Nat. Commun.* **6,** 8018 (2015).

5. Higasa, K. *et al.* Human genetic variation database, a reference database of genetic variations in the Japanese population. *J. Hum. Genet.* **61,** 547-553 (2016).

6. Narahara, M. *et al.* Large-scale East-Asian eQTL mapping reveals novel candidate genes for LD mapping and the genomic landscape of transcriptional effects of sequence variants. *PLoS One* **9,** e100924 (2014).

7. Miki, A. *et al.* Genome-wide association study to identify a new susceptibility locus for central serous chorioretinopathy in the Japanese population. *Investig. Opthalmology Vis. Sci.* **59,** 5542 (2018).

8. Schellevis, R. L. *et al.* Role of the complement system in chronic central serous chorioretinopathy: a genome-wide association study. *JAMA Ophthalmol.* (2018). doi:10.1001/jamaophthalmol.2018.3190
