# Supplementary material for: Genome-wide association analyses identify two susceptibility loci for pachychoroid disease central serous chorioretinopathy
Source: Commun Biol. 2019 Dec 12;2:468. doi: 10.1038/s42003-019-0712-z (PMC6908630; doi:10.1038/s42003-019-0712-z)
Supplement: Supplementary file 2 — Description of Additional Supplementary Files [file 42003_2019_712_MOESM2_ESM.pdf]

Description of Supplementary Data

Supplementary Data 1. Top 100 SNPs in the discovery GWAS.

Supplementary Data 2. The source data about the expression of *TNFRSF10A* and *GATA5* in the adult human retina and RPE. These data was explored in the eyeintegration database (https://eyeintegration.nei.nih.gov/, v1.01. accessed 10 April 2019). The database lists the expression levels of genes given in transcripts per million.
