# Supplementary material for: Genome-wide association analyses identify two susceptibility loci for pachychoroid disease central serous chorioretinopathy
Source: Commun Biol. 2019 Dec 12;2:468. doi: 10.1038/s42003-019-0712-z (PMC6908630; doi:10.1038/s42003-019-0712-z)
Supplement: Supplementary file 5 — Reporting summary [file 42003_2019_712_MOESM5_ESM.pdf]

nature research

Corresponding author(s): Masahiro Miyake

Last updated by author(s): Nov 8, 2019

Reporting Summary

Nature Research wishes to improve the reproducibility of the work that we publish. This form provides structure for consistency and transparency in reporting. For further information on Nature Research policies, see Authors & Referees and the Editorial Policy Checklist.

Statistics

For all statistical analyses, confirm that the following items are present in the figure legend, table legend, main text, or Methods section.

n/a	Confirmed	
☐	☒	The exact sample size (*n*) for each experimental group/condition, given as a discrete number and unit of measurement
☐	☒	A statement on whether measurements were taken from distinct samples or whether the same sample was measured repeatedly
☐	☒	The statistical test(s) used AND whether they are one- or two-sided<br>*Only common tests should be described solely by name; describe more complex techniques in the Methods section.*
☐	☒	A description of all covariates tested
☐	☒	A description of any assumptions or corrections, such as tests of normality and adjustment for multiple comparisons
☐	☒	A full description of the statistical parameters including central tendency (e.g. means) or other basic estimates (e.g. regression coefficient) AND variation (e.g. standard deviation) or associated estimates of uncertainty (e.g. confidence intervals)
☐	☒	For null hypothesis testing, the test statistic (e.g. *F*, *t*, *r*) with confidence intervals, effect sizes, degrees of freedom and *P* value noted<br>*Give P values as exact values whenever suitable.*
☐	☒	For Bayesian analysis, information on the choice of priors and Markov chain Monte Carlo settings
☐	☒	For hierarchical and complex designs, identification of the appropriate level for tests and full reporting of outcomes
☐	☒	Estimates of effect sizes (e.g. Cohen's *d*, Pearson's *r*), indicating how they were calculated

Our web collection on statistics for biologists contains articles on many of the points above.

Software and code

Policy information about availability of computer code

Data collection	Publicly available databases, Genotype-Tissue Expression (GTEx) Portal, eyeintegration database (v1.01), The OCULAR TISSUE DATABASE, Integrative Japanese Genome Variation Database (ver 3.5K JPN), Human Genetic Variation Database and 1000 Genome project (Phase 3), were used to collect data.
Data analysis	R ver 3.5.2, PLINK ver. 2.0, GCTA ver. 1.25.3, Michigan imputation server and VEGAS2pathway ver2 were used for data analysis

For manuscripts utilizing custom algorithms or software that are central to the research but not yet described in published literature, software must be made available to editors/reviewers. We strongly encourage code deposition in a community repository (e.g. GitHub). See the Nature Research guidelines for submitting code & software for further information.

Data

Policy information about availability of data

All manuscripts must include a data availability statement. This statement should provide the following information, where applicable:

- Accession codes, unique identifiers, or web links for publicly available datasets
- A list of figures that have associated raw data
- A description of any restrictions on data availability

Top SNPs (n=100) in the discovery GWAS can be available as Supplementary Data 1. The source data about the expression of genes in the adult human retina and RPE can be available as Supplementary Data 2. The complete GWAS summary data can be visualised here: https://figshare.com/articles/CSC_control_PC3_assoc_logistic/11136047. The datasets generated during the current study are also available from the corresponding author on reasonable request.

Field-specific reporting

Please select the one below that is the best fit for your research. If you are not sure, read the appropriate sections before making your selection.

☒ Life sciences ☐ Behavioural & social sciences ☐ Ecological, evolutionary & environmental sciences

For a reference copy of the document with all sections, see nature.com/documents/nr-reporting-summary-flat.pdf

Life sciences study design

All studies must disclose on these points even when the disclosure is negative.

Sample size

Consecutive 610 Japanese CSC Patients who visited Kyoto University Hospital from 2004 to 2018 were included in the discovery analysis. Replication analysis were performed using 3 independent Japanese and European cohort, and 1546 CSC patients and 13029 controls were included in total.
We revealed that this one-stage GWAS has 87% statistical power, indicating the sufficient power to demonstrate the presence of an association (CaTS software, http://csg.sph.umich.edu//abecasis/CaTS/index.html. Following values were used to estimate the power; Cases=610, controls= 2850, significant level = 0.000001, Disease Model = Multiplicative, prevalence = 0.0001, Disease Allele Frequency = 0.3, Genotype Relative Risk = 1.5).

Data exclusions

The dataset of patients were fixed before analysis, and no data were excluded.

Replication

Three stage replication analysis from three independent cohort consist of Japanese and European were performed. In all stages, the associations of TNFRSF10A rs13278062 and GATA5 rs6061548 with CSC occurrence were consistently replicated, and meta-analysis revealed that both SNPs showed genome wide significant level of association.

Randomization

Three principal components were used as covariates in the discovery GWAS, and we got an acceptable control; the genomic inflation factor lambda (λGC) was 1.157).

Blinding

Diagnosis and genotyping were independently performed;
Initially, retina specialists diagnosed and recruited CSC patients who visited Kyoto University without any genotype information. All patients who were diagnosed as CSC were genotyped and analyzed.
In the replication stages, CSC patients were recruited from other institutes. The diagnosis was made by ophthalmologists at each institute without any genotype information. All patients who were diagnosed as CSC were genotyped and analyzed.

Reporting for specific materials, systems and methods

We require information from authors about some types of materials, experimental systems and methods used in many studies. Here, indicate whether each material, system or method listed is relevant to your study. If you are not sure if a list item applies to your research, read the appropriate section before selecting a response.

Materials & experimental systems

n/a	Involved in the study	
☒	☐	Antibodies
☒	☐	Eukaryotic cell lines
☒	☐	Palaeontology
☒	☐	Animals and other organisms
☐	☒	Human research participants
☒	☐	Clinical data

Methods

n/a	Involved in the study	
☒	☐	ChIP-seq
☒	☐	Flow cytometry
☒	☐	MRI-based neuroimaging

Human research participants

Policy information about studies involving human research participants

Population characteristics

In all stages, cases are CSC patients and controls are healthy subjects.
Characteristic of participants are as follows;

In the discovery GWAS: One-hundred thirty one (21%) of 610 CSC patients were female and the mean age of all patients was 55.4 years. Healthy Japanese controls consist of cataract patients from 7 institutes (n=1656) and general Japanese from the Aichi Cancer Center Research Institute (n = 1,194). Detailed characteristics of controls are described elsewhere (Nat. Genet. 2015 Apr;47 (4): 387-92. and Hum Mol Genet. 2013 Dec 20;22(25):5288-94).

In the replication stage using Kobe dataset: the mean age of 137 CSC patients was 49.4 years, and 23 of them were female as described elsewhere. The mean age of controls was 63.1 years, and 297 of them were female as described elsewhere.(Investig. Opthalmology Vis. Sci. 59, 5542 (2018).)

In the European replication stage: the median age of CSC patients was 51 years, and 420 of them were male. The median age of CSC patients was 52 years, and 1630 of them were male as described elsewhere (JAMA Ophthalmol. (2018). doi:10.1001/jamaophthalmol.2018.3190).

Recruitment

In the discovery GWAS, consecutive 610 Japanese CSC Patients who visited Kyoto University Hospital and assigned to this study were recruited. The diagnosis of CSC petients were performed by 2 independent retina specialists. Control samples (n=2850) were recruited from 8 institutes as shown in SI. Though detailed ophthalmic examinations were not performed for healthy Japanese from the Aichi Cancer Center Research Institute (n = 1,194), all other 1,656 samples were confirmed not to have exfoliation syndrome, macular degeneration, or glaucoma, as described elsewhere.

In the first replication stage, CSC patients (n = 278) were recruited from all over Japan (Kyoto University Hospital, Kagawa University Hospital, Yamanashi University Hospital, and the Fukushima Medical University Hospital). The diagnosis was made by ophthalmologists at each institute. Control samples were recruited from publicly available population-based databases and cataract patients in Yokohama City University (n=5499, in total).

In the second replication stage, the dataset from Kobe University was used. As described elsewhere, individuals with idiopathic CSC (n=137) recruited at Kobe University Hospital and population-based volunteers (n=1153) recruited by Kyushu University were used.

In the third replication stage, European patients with chronic CSC (n=521) recruited from Radboud University Medical Centre, University Hospital of Cologne, and Leiden University Medical Center were included. Controls (n=3577) were obtained from the Nijmegen Biomedical Study, a population-based survey conducted by the Department for Health Evidence and the Department of Laboratory Medicine of the Radboudumc.

Ethics oversight

All procedures adhered to the tenets of the Declaration of Helsinki. The Institutional Review Board and the Ethics Committee of each participating institute approved the respective study protocols. All patients were fully informed of the purpose and procedures of the study, and written consents were received from all patients prior to their participation in the study.

Note that full information on the approval of the study protocol must also be provided in the manuscript.
